# The COVID-19 pandemic behind bars: Experimental evidence showing higher support for decarceration when framed as risk to correctional staff

**DOI:** 10.1016/j.ssmph.2022.101218

**Published:** 2022-08-28

**Authors:** Erin J. McCauley

**Affiliations:** Department of Social and Behavioral Sciences, University of California, San Francisco, USA

**Keywords:** Incarceration, COVID-19, Experiment, Decarceration, Public support

## Abstract

This study examined the effect of framing COVID-19 spread in correctional facilities as impacting imprisoned individuals or impacting correctional staff on public support for decarceration. I employed an experiment in the 2021 Empire State Poll (n = 765) in which participants were randomly assigned to a treatment condition, which highlighted information about the number of COVID-19 cases among imprisoned individuals, or a control condition, which highlighted correctional staff instead. Participants reported how supportive or unsupportive they are of releasing imprisoned individuals to curb the spread of COVID-19. Overall, 35% of New Yorkers supported decarceration. A higher percentage of respondents supported decarceration when the impact on correctional staff was highlighted (40%) relative to imprisoned individuals (31%). There was also higher support among non-Hispanic Black (54%) and Hispanic (51%) participants relative to non-Hispanic White (28%) participants. Within racial/ethnic groups support for decarceration was higher when the impact on correctional staff was highlighted among non-Hispanic Whites, Hispanics, and those of another race, but not among non-Hispanic Blacks where support for decarceration was higher when the impact on imprisoned individuals was highlighted. Inferential analysis using log binomial regression found that the association between treatment condition assignment and support for decarceration was not significant. Public health practitioners and policy makers should consider leveraging the higher support associated with concerns over the health and wellbeing of correction staff found among some racial/ethnic groups to fight the COVID-19 pandemic.

## Highlights

• Thirty five percent of New Yorkers support decarceration to protect public health.
• Experiment shows higher support for decarceration when COVID threatens staff.
• There is lower support for decarceration when COVID threatens imprisoned people.
• Racial variation in support for decarceration, with lowest support among Whites.
• Support for decarceration higher if framed as risk to imprisoned only among Blacks.

## 1 Introduction

The rapid expansion of the criminal legal system in the United States (US) over the last half-century has had implications for population health and contributed to the widening of health inequity within in the US and between the US and peer nations (Wildeman & Wang, 2017). The US has the highest incarceration rate in the world and the highest number of confirmed COVID-19 cases in the world (Macmadu et al., 2020). In correctional facilities, traditional strategies to reduce the spread of COVID-19 are often not possible. Correctional facilities are crowded congregate living areas in which social distancing is difficult and there is controlled access to personal protective equipment (PPE) and soaps and sanitizers (Macmadu & Brinkley-Rubinstein, 2021; Macmadu et al., 2020). This makes smart decarceration, or the intentional reduction of the incarcerated population, central in the effort to curb COVID-19 spread (Data For Progress, 2020; Macmadu & Brinkley-Rubinstein, 2021).

The US experienced modest reductions in the incarcerated population early in the COVID-19 pandemic; however, reductions in the incarcerated population were controversial (Gaines, 2021; Zumer, 2020) and short-lived (Prison Policy Initiative, 2021). Importantly, these reductions came through decreased admissions instead of releases and, in some cases, exacerbated racial inequality (Data For Progress, 2020; Hager, 2021). Thus, understanding public support for decarceration is a pivotal next step. This study employed a survey-based experiment, fielded in March and April of 2021, to examine how framing the health risks of COVID-19 for imprisoned individuals (treatment condition) and correctional staff (control condition) shapes public opinion on decarceration in New York (NY) state. NY is more progressive than many other states on both criminal justice and COVID-19 mitigation policies.

### 1.1 Prevalence of US Incarceration

The US has the highest incarceration rate in the world (Prison Policy Initiative, 2021). Importantly, incarceration in the US is also highly unequal across social groups with an especially concentrated risk of contact with the criminal legal system among Black Americans (Alexander, 2010). Black Americans experience disproportionately high rates of incarceration (Carson, 2020; Mauer & King, 2007) and have higher rates of incarceration in familial and social networks (Enns et al., 2019; Lee & Wildeman, 2021; Wildeman, 2009). There is evidence that excessive incarceration in the Black community in the US is a result of structural racism and the lasting legacy of slavery (Alexander, 2010). A Black individual has 1.8 times the odds of being convicted and serving time than a White individual for the same offense (Camplain et al., 2020) and a meta-analysis found that non-White suspects were more likely to be arrested than White suspects for similar crimes (Kochel et al., 2011). The racial disparity in the risk of experiencing incarceration, coupled with the poor consequences for health (Massoglia & Pridemore, 2015), have positioned mass incarceration as an important yet lesser-acknowledged contributor to racial disparities in health in the US (Macmadu et al., 2020; Wildeman & Wang, 2017).

### 1.2 COVID-19 and correctional facilities

In the context of the COVID-19 pandemic, the individual and population-level health risks of incarceration have increased (Akiyama et al., 2020; Wang et al., 2020). Among the incarcerated population in the US, the confirmed COVID-19 case rate is five times greater and the mortality rate is three times greater than in the general population (Marquez et al., 2021). According to the COVID Prison Project, as of April 19th, 2022 there have been more than 580 thousand positive COVID-19 tests among imprisoned individuals and nearly three thousand have died of COVID-19 (Brinkley-Rubinstein & Nowotny, 2022). Incarcerated individuals in the US are at a high risk of complications from COVID-19 due to their elevated risk of underlying medical complications (Macmadu et al., 2020; Nowotny et al., 2020). Moreover, incarcerated individuals are at a higher risk of contracting COVID-19 due to the confinement conditions, including overcrowding, congregate living, and limited access to resources to mitigate the risk of infection (such as soaps, sanitizers, and medical care) (Akiyama et al., 2020; Macmadu et al., 2020; Nowotny et al., 2020; Saloner et al., 2020). The New York Times found that 40 of the largest 50 outbreaks in the US occurred in correctional facilities (Macmadu et al., 2020) and there was inconsistent use of COVID-19 testing in correctional facilities in the early days of the pandemic (Lemasters et al., 2020).

Correctional staff also face elevated risks of COVID-19 exposure. According to the Covid Prison Project, as of April 19th, 2022, there have been nearly 200 thousand positive COVID-19 tests among correctional staff and 277 had died of COVID-19 (Brinkley-Rubinstein & Nowotny, 2022). The rate of COVID-19 infection among correctional staff is higher than in the general population, approaching but not surpassing the risk for imprisoned individuals (Nowotny et al., 2021; Towers et al., 2022).

### 1.3 Mass incarceration & COVID-19 in NY state

This study occurred in March and April of 2021 in NY state, which was among the first states in the US to experience a serious outbreak of COVID-19, and coincided with the roll out of the COVID-19 vaccine. Only 22% of states prioritized vaccinating incarcerated individuals in Phase 1 of vaccination efforts even though states recognized correctional facilities as areas of high risk (Strodel et al., 2021). Nearly half of the states did prioritize prison and jail employees in Phase 1 efforts (Strodel et al., 2021). Although data on vaccinations among imprisoned individuals and staff are available for nearly 30 states, these data are not reported by NY. News coverage from 2021, when this study's data collection occurred, suggest that the vaccination rate among imprisoned individuals lagged behind the general population in NY (Associated Press, 2021; Russell, 2021). In NY universal eligibility for COVID-19 vaccines was announced in April of 2021, during the survey fielding.

Both imprisoned individuals and correctional staff in NY had elevated risks of contracting COVID-19 at the time of the study. Researchers found that the risk of COVID-19 was three times greater among NY correctional staff than the general population (Nowotny et al., 2021). According to the Covid Prison Project, by April 1st, 2021, there were 5,885 positive COVID-19 cases among imprisoned individuals in NY (Brinkley-Rubinstein & Nowotny, 2022), equating to 187 positive COVID-19 tests per 1,000 individuals. Similar to the national trend, the number of deaths was considerably higher among the imprisoned population in NY compared to correctional staff, despite a similar number of infections both groups. In fact, the case fatality rate was nearly two and half times greater among the imprisoned population than the correctional staff population in NY (Brinkley-Rubinstein & Nowotny, 2022).

### 1.4 The need for decarceration

In response to this combination of crises, public health scholars and practitioners have issued an urgent call for decarceration (i.e., reducing the number of incarcerated individuals) (Akiyama et al., 2020; Hawks et al., 2020; Macmadu & Brinkley-Rubinstein, 2021; Macmadu et al., 2020; Sivashanker et al., 2020). This is especially important given likely vaccine hesitancy among imprisoned individuals and correctional staff, as well as turnover rates in correctional settings (Barsky et al., 2021). Experts have argued that, in addition to reducing the risk of COVID-19, decarceration efforts will also have the added benefit of reducing the burden of overcrowding and underfunding and would begin the work of deconstructing the larger legacy of structural racism (Franco-Paredes et al., 2021; Macmadu et al., 2020). Additionally, there is evidence that decarceration efforts lead to a reduced risk of contracting COVID-19 (Macmadu et al., 2020) in correctional settings and reduced COVID-19 growth rates in the community (Barsky et al., 2021). Further, decarceration has not been linked with increased crime (American Civil Liberties Union, 2020.

Calls for large-scale decarceration as a strategy to curb the spread of COVID-19 have been unheeded. A lack of discretion for prison releases has been a barrier to decarceration (Reitz, 2020). However, in the early months of the COVID-19 pandemic expedited releases were made more accessible. There have been moderate reductions in the correctional population, however, these have largely resulted from reductions in admissions and not releases (Data For Progress, 2020). For the state prison population, the number of monthly releases for reporting states was lower in 2021 than the two years prior (Prison Policy Initiative, 2021). For the jail population, there was originally a 25% reduction in the incarcerated population in the months following the COVID-19 outbreak in the US (Prison Policy Initative, 2021). However, these reductions largely resulted from ad hoc efforts and rule adjustments which were not sustained. Nearly 30% of jails in the US have a larger incarcerated population now than in March of 2020 (Prison Policy Initiative, 2021). This pattern is also true in NY. Rikers Island, which experienced the first COVID-19 outbreak in a correctional facility, similarly saw a dramatic decrease in population which has slowly been undone, returning to pre-COVID-19 levels and even experiencing a nearly 20% increase in the pre-trial detention population during the pandemic (Prison Policy Initiative, 2021).

### 1.5 Sustained decarceration and the role of public support

One potential reason for the lack of sustained decarceration is how influential public opinion is for criminal law relative to other types of law (Barkow, 2019). State policies generally follow and reflect public opinion on policy matters, especially concerning morality-related policies (Lewis & Oh, 2008). This is especially true for criminal legal policy, where public opinions and media coverage tends be driven by the worst outliers (Barkow, 2019). Incarceration rates are not driven by crime rates, but rather by jurisdictional-policies (Clear, 2021). The lack of insulation protecting criminal legal policy from the political winds of politicians, voters, and the media has been a persistent barrier to criminal legal reform (Barkow, 2019).

In the case of decarceration, public support may be a necessary step toward sustained change. Indeed, some have theorized that participation by politicians without fear of public blow-back at varying levels of government is imperative in creating a context in which large-scale reforms leading to sustained decarceration are possible (Barkow, 2019; Gartner et al., 2011). One study found that public opinion on punitiveness was a primary driver behind the growth of mass incarceration and that if public support for punitiveness had held at the level of the 1970s instead of growing exponentiality there would have been 20% lower incarceration in the decades that followed (Enns, 2014). Indeed, one of the largest reductions in incarceration in US history occurred in California's state prison population and is thought to be attributable to political buy-in throughout the state and public support for the effort (Gartner et al., 2011). Others have argued that changing public perceptions of incarceration is one of the necessary conditions for smart decarceration (Epperson & Pettus-Davis, 2015).

Unfortunately, incarcerated individuals and communities disproportionately affected by mass incarceration have diminished political power (Thompson, 2011). Incarcerated individuals in most states are stripped of their right to vote while incarcerated (with the exception of Maine, Vermont, and Washington D.C.) and in many states formerly incarcerated individuals lose their right to vote for sustained periods (Gartner et al., 2011). To achieve decarceration, there must be broad public support for the effort to elect politicians who are supportive of policy reform. Currently, decarceration has lower public support than other proposed measures to control COVID-19 in correctional facilities. A poll by Data for Progress with likely voters found that there is 52% support for releasing incarcerated individuals that do not pose a serious physical safety risk to the community, compared to 82% support for providing soap, hand sanitizer, and medical care to incarcerated individuals (Data For Progress, 2020).

### 1.6 This study

The objective of this study was to examine how framing the risk of COVID-19 to imprisoned individuals or correction staff would shape public support for decarceration efforts to limit the spread of COVID-19. A robust body of literature has shown that framing and communication of facts can influence public opinion (e.g., Shapiro & Bloch‐Elkon, 2008; Slothuus, 2010). This is particularly true is the framing or facts resonate with preexisting beliefs or personal experience (Slothuus, 2010). It is likely that the public will have different levels of support for decarceration when framed as affecting imprisoned individuals or correctional staff for several reasons, but it is unclear which group will garner higher support. The number of positive COVID-19 tests among imprisoned individuals in NY was four and quarter times greater than among correctional staff. This larger number could represent a larger threat to public health and safety, thereby generating higher support. However, public opinion of correctional officers is generally more favorable than public opinion of imprisoned individuals. The public views correctional work as more prestigious than other blue collar employment, and consider the work to be “useful”, “meaningful”, and to entail a lot of “responsibility” (Sundt, 2009). Alternatively, incarcerated individuals are a stigmatized group. A meta-analysis of public opinions toward formerly incarcerated individuals found there are only minor differences across groups and communities, suggesting uniformly negative attitudes (Rade et al., 2016). These negative views may lead correctional staff to garner higher support. This study employed an experimental design with NY state residents to test how framing COVID-19 as affecting imprisoned individuals (treatment condition) or correctional staff (control condition) shapes support decarceration to curb the spread of COVID-19.

## 2 Materials and methods

This study employed an experimental design to evaluate how exposure to information about the number of confirmed COVID-19 cases among correctional staff or imprisoned individuals shape public support for decarceration. The study was approved by the Cornell IRB. The experiment was fielded as part of The Empire State Poll (ESP), a statewide representative survey in NY which is conducted by The Cornell Survey Research Institute. The experiment was submitted and selected by The Cornell Survey Research Institute as part of the omnibus model allowing the study of special topics through a competitive application process. The ESP is conducted annually with NY residents who are at least 18 years of age. The ESP uses a dual-frame random digit dial sample covering both cellular and landline telephones and an additional sampling frame to cover cell phones of NY residents who have out of state cell phone numbers (11%). Every household with a telephone had an equal chance of inclusion in the ESP, and once a household was selected every adult in the household had an equal chance of being selected. Interviews were conducted through a Computer-Assisted Telephone Interviewing software system. The average interview length was 25 min. For more information about the sampling methodology, see the ESP methodology report (Sharab, 2020). The data were collected between March and April of 2021 and the total sample consisted of 800 New Yorkers.

Participants were randomly assigned to the treatment condition, which heard “Nearly 360 thousand people in prison in the US have tested positive for COVID-19”, or the control condition, which heard “Nearly 85 thousand people who work in prisons in the US have tested positive for COVID-19”. Then all participants heard “Allowing the virus to spread in prison puts the prison population, the employees of the prison, and the health of communities at risk.” Participants were asked, “Would you support, oppose, or neither support not oppose, releasing people from prison to reduce the spread of COVID-19?”. Participants selected an answer from 1) strongly support, 2) somewhat support, 3) neither support nor oppose, 4) somewhat oppose, and 5) strongly oppose. Participants could also respond that they did not know or refused to answer.

The number of COVID-19 cases were taken from the Covid Prison Project (Brinkley-Rubinstein & Nowotny, 2022). The cases were presented as raw numbers for ease of interpretation by the public and to demonstrate the scope of the threat to public health. While case rates would allow easier comparison between the level of COVID-19 among imprisoned individuals and correctional staff, participants were only exposed to one condition or the other.

The independent variable is if participants were in the treatment or control condition. The dependent variable is if respondents support decarceration, measured as a binary variable. The inferential analysis included covariates for participant race (non-Hispanic White, non-Hispanic Black, Hispanic, and other) and gender (male, female). Participants with missing data on the outcome variable, support for decarceration, were dropped from the sample (n = 35, 4.4%).

The Cornell Survey Research Institute developed survey weights for use in analyses intended to generalize findings to the population of New York, as well as by upstate and downstate residents, using an iterative raking process. This process uses population proportions for downstate and upstate New York regions, gender, age, Black population, Hispanic population, education, household income, and party affiliation. These weights are based on the 2018 American Community Survey 5-year estimates and New York State Board of Elections data. A maximum of four iterations were completed. Additional details about the development of weights can be found in the ESP methodology report for 2020, which used the same methodology (Sharab, 2020).

### 2.1 Analyses

First, overall support for decarceration among NY residents was examined. I present the weighted proportion of participants who supported decarceration for the entire sample and disaggregated by racial-ethnic group and gender. Then, this study examined the proportion of NY residents who supported decarceration among those assigned to the treatment and control conditions for the full sample and disaggregated by racial-ethnic group and gender. Last, log binomial regression was used to estimate the risk ratio of supporting decarceration for the treatment condition relative to the control condition in a bivariate model and one adjusting for racial-ethnic group and gender. Weights were used in all analyses to generate estimates which are representative of NY state.

## 3 Results

Thirty-five percent of the New Yorkers supported decarceration to reduce the spread of COVID-19 in correctional facilities (confidence interval, or CI = 0.29, 0.42), as seen in Table 1. There was variation in support for decarceration across racial and ethnic groups, with the highest level of support for decarceration among Non-Hispanic Black (54%, CI = 0.36, 0.72) and Hispanic (51%, CI = 0.34, 0.68) populations and the lowest level of support among Whites (28%, CI = 0.21, 0.35). There was also higher support for decarceration among men (37%, CI = 0.28, 0.45) relative to women (34%, CI = 0.25, 0.43), although this difference was modest. Of those in the treatment condition, meaning they were exposed to the number of individuals in prison who tested positive for COVID-19, 31% supported decarceration (CI = 0.23, 0.39), as seen in Table 2. Of those in the control condition, meaning they were exposed to the number of correctional staff who tested positive for COVID-19, 40% supported decarceration (CI = 0.31, 0.49). This is a difference of nine percentage points in support of decarceration between the experimental conditions.

**Table 1:** Descriptive results showing the proportion in support of decarceration across groups.

	Proportion Supporting Decarceration	95% CI	*N*
Full Sample	0.35	(0.29, 0.42)	765
Non-Hispanic White	0.28	(0.21, 0.35)	497
Non-Hispanic Black	0.54	(0.36, 0.72)	93
Hispanic	0.51	(0.34, 0.68)	89
Other	0.29	(0.11, 0.47)	64
Male	0.37	(0.28, 0.45)	381
Female	0.34	(0.25, 0.43)	375

*Notes.* CI is confidence interval.

**Table 2:** Descriptive results showing the proportion in support of decarceration by group.

Proportion Supporting Decarceration; proportion (95% CI) [*n*]	Treatment Condition	Control Condition	Difference by Condition
Full Sample	0.31 (0.23, 0.39) [366]	0.40 (0.31, 0.49) [399]	−0.9
Non-Hispanic White	0.25 (0.14, 0.35) [237]	0.31 (0.22, 0.40) [260]	−0.06
Non-Hispanic Black	0.65 (0.45, 0.85) [55]	0.36 (0.14, 0.58) [38]	0.29
Hispanic	0.38 (0.20, 0.56) [57]	0.73 (0.53, 0.93) [32]	−0.35
Other	0.10 (−0.02, 0.22) [38]	0.38 (0.02, 0.74) [26]	−0.28
Male	0.33 (0.23, 0.44) [202]	0.41 (0.27, 0.54) [179]	−0.08
Female	0.30 (0.16, 0.43) [191]	0.40 (0.28, 0.52) [184]	−0.10

*Notes.* Treatment condition refers to those who were exposed to the number of imprisoned individuals who tested positive for COVID-19. The control condition refers to those who were exposed to the number of correctional staff who tested positive for COVID-19. CI is confidence interval.

There were racial-ethnic and gender differences in support of decarceration within the treatment and control conditions, as well as in the difference in support between the treatment and control condition, as seen in Table 2. There was higher support for decarceration among those in the control condition for non-Hispanic Whites (diff = 0.06), Hispanics (diff = 0.35), and those of another race (diff = −0.28). There was also higher support for decarceration among those in the control condition for Males (diff = 0.08) and Females (diff = 0.10). This trend was not found for non-Hispanic Black participants, among whom support for decarceration was substantially higher among those in the treatment condition (65%, CI = 0.45, 0.85) relative to the control condition (36%, CI = 0.14, 0.58). Overall, the highest support for decarceration in the treatment condition was among Non-Hispanic Blacks (65%, CI = 0.45, 0.85) and in the control condition was among Hispanics (73%, CI = 0.53, 0.93), with Whites having low support for decarceration in both conditions (25% in the treatment condition, CI = 0.14, 0.35) and 31% in the control condition, CI = 0.22, 0.40).

The log binomial regression, as seen in Table 3, found that the risk of the treatment condition group supporting decarceration were 0.78 times the risk of the control condition (*p* = 0.17, CI = 0.55, 1.11), but this association was not statistically significant. This means that exposure to information about COVID-19 among imprisoned individuals or correctional staff was not significantly associated with support for decarceration. In the multivariate model, which adjusts for race/ethnicity and gender, treatment condition assignment was associated with 0.76 times the risk of supporting decarceration relative to control condition assignment (*p* = 0.17). The CI for this estimate ranged from 0.52 times the risk to 1.11 times the risk. This relationship was again not statistically significant. When examining race/ethnicity, non-Hispanic Black participants had 2.01 times the risk of supporting decarceration (p < 0.00, CI = 1.34, 3.01) and Hispanic participants had 2.08 times the risk of supporting decarceration (p < 0.01, CI = 1.35, 3.20) relative to non-Hispanic White participants. Being of another race relative to non-Hispanic White and gender was not significantly associated with the risk of supporting decarceration.

**Table 3:** Results of log binomial regressions estimating risk ratios of support for decarceration using a bivariate model and a model adjusting for race and gender.

	Bivariate Model			Multivariate Model		
	Risk Ratio (std. err.)	P-Value	95% C.I.	Risk Ratio (std. err.)	P-Value	95% C.I.
Treatment Condition	0.78 (0.14)	0.169	(0.55, 1.11)	0.76 (0.15)	0.166	(0.52, 1.11)
Race						
NH Black	–	–	–	2.01** (0.42)	0.001	(1.34, 3.02)
Hispanic	–	–	–	2.08** (0.46)	0.001	(1.35, 3.20)
Other	–	–	–	0.94 (0.43)	0.892	(0.38, 2.33)
Male				1.20 (0.21)	0.302	(0.85, 1.68)
Constant	0.40*** (0.05)	0.000	(0.46, 0.97)	0.23*** (0.05)	0.000	(0.20, 0.39)
*N*	765			739		

*Notes.* Treatment condition refers to those who were exposed to the number of imprisoned individuals who tested positive for COVID-19. The control condition refers to those who were exposed to the number of correctional staff who tested positive for COVID-19. Std. err. is standard error, NH is non-Hispanic, for race the comparison group is non-Hispanic Whites. Weights are used.

## 4 Discussion

In NY in the spring of 2021, more than a third of residents supported decarceration to reduce the spread of COVID-19. This is lower than the level of support captured by other studies (Data For Progress, 2020), which may be a result of differences in how the question was presented to participants or the population itself which is limited to NY state in this study. Both the geographic and temporal context of this study are important to note, as NY was among the first locations in the US to have a large and deadly COVID-19 outbreak, there were dramatic disparities in COVID-19 case rates and case mortality rates, and these data were collected several months after the 2020 racial justice protests. These contexts spurred a renewed conversation about the US criminal legal system and racial and ethnic disparities in health. This study found evidence of variation in support for decarceration between the experimental conditions and between racial-ethnic groups. These differences can inform the work of public health practitioners and policy makers.

As the treatment condition contained a higher total number of infections in the correctional setting than the control condition, it would be logical that support for decarceration would be higher in the treatment condition. The only group for whom this was true was non-Hispanic Blacks, who had higher support for decarceration when framed as a risk to imprisoned individuals. In the combined full sample and for the other racial and ethnic groups, I found the opposite trend. The descriptive results suggest that framing COVID-19 as a threat to the wellbeing of correctional staff improves public support in NY among non-Hispanic Whites, Hispanics, and people of another race, even though the total number of infections is smaller. This is more in line with the hypothesis that the stigma of criminal legal involvement and the public's negative attitudes toward incarcerated individuals (Rade et al., 2016) may lead to lower support for decarceration when framed as a risk to imprisoned people.

While the COVID-19 pandemic presents a unique threat to health, correctional staff face numerous health risks as a result of their employment beyond the pandemic. A recent systematic review found consistent and robust evidence of elevated rates of post-traumatic stress disorder, depression, and anxiety among correctional officers, compared to both similar occupations and the general population (Regehr et al., 2021). Additionally, the risk of suicide among correctional officers is higher than other groups (Stack & Tsoudis, 1997). For example, one study found that the risk of suicide was 2.3 times greater than non-correctional law enforcement officers and 2.5 times greater than similarly aged males in the general population (New Jersey, 2009). Given these other health risks, future research should explore if the health risks of correctional settings to the staff who work in them may have the power to drive decarceration policy, even beyond the context of a pandemic.

Second, the results of this study suggest the potential of differential messaging to promote support for decarceration among various racial and ethnic groups, at least in the NY context. Not surprisingly, support was higher among non-Hispanic Black (54%) and Hispanic (51%) participants than among non-Hispanic White residents (28%), populations which both have disproportionate involvement in and exposure to the criminal legal system (Enns et al., 2019; Mauer & King, 2007).

More interesting, however, is the variation in the level of support and difference in that level between experimental conditions among non-Hispanic Black and Hispanic participants. For non-Hispanic Black participants, support was substantially higher in the treatment condition (65%) than the control condition (36%). This may be a result of the overrepresentation of Black Americans in the criminal legal system and the resonance of this frame with the preexisting beliefs of this group. Black Americans are incarcerated in state and federal prisons at five times the rate of non-Hispanic White Americans (Carson, 2020). Black Americans are also disproportionately exposed the criminal legal system. For example, 60% of Black Americans have had an immediate family member incarcerated (Enns et al., 2019) and correctional staff are disproportionately Black (Bureau of Prisons, 2022). Research on framing effects have found that frames are especially powerful for shaping public opinion when resonant with a public's preexisting beliefs (Slothuus, 2010), which can stem from personal experience.

For Hispanic participants, however, the opposite was true. Hispanics had lower support for decarceration in the treatment condition (38%) than the control condition (73%). This difference is notable, as Hispanics are also overrepresented in the criminal legal system, with an imprisonment rate that is two and half times greater than that of non-Hispanic Whites (Carson, 2020) and with nearly half of Hispanics having had an immediate family member incarcerated (48%) (Enns et al., 2019). While support for decarceration was higher among Hispanics in the control condition, support among both conditions was still higher among Hispanics than non-Hispanic Whites in either condition.

Taken together, policy makers and health practitioners responding to the calls of experts to promote decarceration (e.g., Macmadu & Brinkley-Rubinstein, 2021; Wang et al., 2020) should considering using differential messages for different racial-ethnic groups. For non-Hispanic White and Hispanic populations, public health practitioners and policy makers should frame the risk of COVID-19 to correctional staff to garner higher support for decarceration. However, a different message emphasizing the risk to imprisoned individuals should be used to garner support for decarceration among non-Hispanic Black populations.

The results of this experiment find higher support for decarceration efforts among those who were exposed to the correctional staff threat framing, at least among some populations and in the context of a largely progressive Northeastern state. This is critical information as decarceration would be beneficial for population health and disparities in health in the COVID-19 era. Decarceration is pivotal in reducing rates of new COVID-19 infections. COVID-19 spread in correctional facilities is tied to community rates (Reinhart & Chen, 2020), and research has shown that spread in the community leads to spread in correctional facilities (LeMasters et al., 2022). Decarceration could be an important tool in managing the pandemic (Franco-Paredes et al., 2021; Leibowitz et al., 2021; Macmadu & Brinkley-Rubinstein, 2021; Data For Progress, 2020; Wang et al., 2020). Decarceration would allow those who are released to be reconnected with families and integrated into communities while allowing greater social distancing and increased access to limited resources (such as PPE and soaps) for those who remain incarcerated (Franco-Paredes et al., 2021).

There has been an emerging public willingness to support decarceration beyond the context of a pandemic (Clear, 2021). It is possible that this aim could be furthered if policy makers and politicians could capitalize on this moment, when the health of incarcerated individuals, correctional staff, and the public are inextricably linked. Building a coalition of support for decarceration long term is important in reducing racial disparities in health and wellbeing. There are extreme racial/ethnic and class disparities in incarceration and incarceration is detrimental to health even outside the context of a pandemic (Federal Bureau of Prisons, 2022; Cloud et al., 2020; Massoglia & Pridemore, 2015; Ranapurwala et al., 2018; Wildeman & Wang, 2017). Taken together, this means that mass incarceration and the criminal legal system contribute to racial health inequity (Wildeman & Wang, 2017) and decarceration should be a public health priority (Advancing Public Health Interventions, 2020). Leveraging the increased support for decarceration resulting from concerns about the welfare of correctional officers in this context can fuel further political will and public support for decarceration, thereby promoting health equity and taking steps to dismantle the criminal legal system (Franco-Paredes et al., 2021).

It is important to note, however, that public support alone is not sufficient context for developing a political landscape where sustained and smart decarceration policy reform is possible (Barkow, 2019). Although the reduction of the incarcerated population in the US has been modest, there has been considerable controversy over the existing efforts (Gaines, 2021; Zumer, 2020). Thousands of individuals were released from federal prisons to home confinement through the CARES Act, but in January of 2021, the Trump Administration released a memo stating that those who were released must return to federal prisons 30 days after the end of the national emergency declaration related to COVID-19 (Federal Bureau of Prisons, 2021). The future of the more than four thousand released individuals remains unclear, with the Federal Bureau of Prisons maintaining that those released will eventually need to return despite public outcry (Gaines, 2021). Furthermore, the slight reductions in the incarcerated population have been unequal, reifying and exacerbating racial disparities in detention (Hager, 2021), emphasizing the need for intentional reductions in the incarcerated population.

Researchers and healthcare providers need to turn their attention to decarceration, educating politicians and the public on the risks of crowded congregate living facilities for contagious diseases and viral infections in the COVID-19 context and beyond, studying the potential benefits of decarceration, and preparing to support formerly incarcerated individuals upon release. Furthermore, the limited initial reductions in incarceration seen at the beginning of the pandemic have largely been reversed, emphasizing the need for additional research to examine how to convert short-term and pandemic related movements toward decarceration into sustained long-term decarceration.

This study focuses on the residents of one state and explores decarceration support due to the number of COVID-19 confirmed infections. Future studies should compare other measures of COVID-19 exposure (such as deaths, the number of cases in local facilities, or information about actual cases which are attributable to correctional facility spread) to get a more nuanced understanding of how framing may affect public support.

It would also be of interest to expand the geographic location in consideration to other regions of the US This is especially true given the political and social context of NY is considerably more progressive in terms of both COVID-19 policies and criminal legal reform than other states. Future research should consider how framing the risk of COVID-19 for incarcerated individuals or correction staff shapes public support for decarceration Southern states, where incarceration rates are the highest in the US and the world (Prison Policy Initative, 2021) and where COVID-19 mitigation policies have had the least support (Kaiser Family Foundation, 2021). It would be informative to examine how concern for the health and wellbeing of correctional staff more broadly could be leveraged to promote decarceration outside of the context of a pandemic.

Another limitation of note is that the weights used in these analyses were designed to generate estimates which are representative of NY state, and not racial/ethnic subgroups (although these groups were oversampled). Thus, the results by racial/ethnic groups should be interpreted with caution.

## 5 Conclusion

The fervent call for decarceration in the U.S to curb the spread of COVID-19 should include a focus on the consequences of unchecked spread for correctional staff, especially for non-Hispanic White and Hispanic populations for whom leveraging the welfare of correctional officers may increase support for decarceration in the NY context. Public support for decarceration will be key in the political effort to achieve it, and in the efforts to reduce racial disparities in COVID-19. Moreover, beginning the process of decarceration would have health and justice promoting consequences that extend beyond the current COVID-19 pandemic (Macmadu et al., 2020; Minkler et al., 2020). Leveraging the risk of COVID-19 to correctional staff could be used by policy makers and practitioners to facilitate the political context needed to implement larger-scale policy reforms to create sustained decarceration. Decarceration must be included in the conversation about COVID-19 and will be an important step in the efforts to reduce racial disparities in health and wellbeing (Macmadu et al., 2020; Minkler et al., 2020).

## Ethics statement

This study was conducted under the oversight of the Cornell Institutional Review Board and the data was collected by the Cornell Survey Research Institute.

## Author statement

EM: Conceptualization; Data curation; Formal analysis; Funding acquisition; Investigation; Methodology; Software; Validation; Writing - original draft; Writing - review & editing.

## Declaration of competing interest

No known conflict of interest.

## Data Availability

The authors do not have permission to share data.
